# A Chapter on Wrinkles

**Published:** 1893-04

**Authors:** 


### A CHAPTER ON WRINKLES.

There is nothing so destroying to the peace of a pretty woman's soul as the discovery of the first wrinkle in her fair face. Gray hairs may be tolerated, for often their framing softens the tints of the complexion and adds new depths and brightness to the eyes that flash beneath them, and many pretty women are never really beautiful until they are crowned with the sheen of silver tresses. The fading tints of a well kept and smooth skin may be concealed by artifices that every wise woman knows, but a wrinkle is an obstinate, disagreeable, aggressive witness, that leaves evidence of age in most unpicturesque language, as convincing as the records of the family Bible, or the testimony of some old friend of your mother's who is always telling every one that you are "just two years older than her Johnny," when perhaps you look ten years younger.

There is no such a thing as conciliating a wrinkle or coaxing it out of sight on occasions, no dressing it up in pretty disguises, gauze and frills; no one ever really admired its curves or wrote sonnets to its beauty; no one ever really longed for its coming or succeeded in banishing it by a cool reception; it comes uninvited and tarries unbidden, and settles more contentedly in its place as you fume and fret over it.

Many remedies for the eradication of wrinkles have been suggested by various writers on the subject of personal beauty, but the best and surest cure for wrinkles is not to get them, for they may be avoided more easily than removed. Wrinkles are not always the signs of age, but often the indices of a poorly-cared-for skin, the nervous temperament of their possessor, the habit of excessive worrying or continuous study, and sometimes of the degeneracy of the race. Italian children of five or six years often have more wrinkles in their little faces than a woman of eighty-five ought to possess.

A skin that is carefully and frequently bathed in warm water and pure soap, and rubbed to a glow all over once each day with soft flannel or the hands, preserves its elasticity and is less susceptible to wrinkles. The modern woman has more cares and perplexities and worries than Cæsar ever dreamed of. If Alexander had had one of the average nineteenth-century servants to manage he would never have sighed for new worlds to conquer, since fresh developments would have awaited him every morning. But these cares and worries are in no way ameliorated by expressing them in the face with countless grimaces and contortions of feature that invariably produce lines. The vivacity and swift-changing play of feature in bright, sparkling girls makes prematurely wrinkled and distracted-looking women. Much of this vivacity and pretty by-play of elevated brows is forced and unnatural, and all the more conducive to wrinkles.

Another habit women have is of contorting their faces into most ludicrous and ugly positions when exposed to the strong sunlight, all of which, by a little thought and effort, can be controlled to a degree.

A very beautiful and youthful-appearing society woman, the preservation of whose skin is remarked upon by her acquaintances, says that whenever she is going out in the evening she prepares her toilet, with the exception of her dress, wrings a wash-cloth out of as hot water as she can bear, smooths it out over her face so it will touch every part of it, and lies with it on her face for half an hour. When she removes it every wrinkle and line have disappeared.

An English lady over fifty asserts that her lack of wrinkles is due to the fact of her having used very hot water all her life, which tightens the skin and smooths out the lines.

Another celebrated beauty attributes her preservation to having never used a wash-cloth or towel on her face, but having always washed it gently with her hand, rinsing it off with a soft sponge, drying it with a soft cloth, and then rubbing it briskly with a flesh-brush. She used castile soap and very warm water every night, with cold water in the morning, and if she were awake late at night, she always slept as many hours in the day as she expected to be awake at night.

Another student of the toilet asserts that she prevents and obliterates wrinkles by rubbing the face towards the nose when bathing it, and Ella Wheeler Wilcox asserts that she can eradicate a permanent wrinkle by the use of almond paste and friction.—*Ex*.

